# Sleep habits and strategies of ultramarathon runners

**DOI:** 10.1371/journal.pone.0194705

**Published:** 2018-05-09

**Authors:** Tristan Martin, Pierrick J. Arnal, Martin D. Hoffman, Guillaume Y. Millet

**Affiliations:** 1 Human Performance Laboratory, Faculty of Kinesiology, University of Calgary, Calgary, Canada; 2 Rhythm, San Francisco, CA, United States of America; 3 Department of Physical Medicine & Rehabilitation, Department of Veteran Affairs, Northern California Health Care System, Sacramento, CA, United States of America; 4 University of California Davis Medical Center, Sacramento, CA, United States of America; 5 Ultra Sports Science Foundation, Sacramento, CA, United States of America; Norwegian University of Science and Technology, NORWAY

## Abstract

Among factors impacting performance during an ultramarathon, sleep is an underappreciated factor that has received little attention. The aims of this study were to characterize habitual sleep behaviors in ultramarathon runners and to examine strategies they use to manage sleep before and during ultramarathons. Responses from 636 participants to a questionnaire were considered. This population was found to sleep more on weekends and holidays (7–8 h to 8–9 h) than during weekdays (6–7 h to 7–8 h; p < 0.001). Work was a mediator of napping habits since 19–25% reported napping on work days and 37–56% on non-work days. There were 24.5% of the participants reporting sleep disorders, with more women (38.9%) reporting sleep problems than men (22.0%; p < 0.005). Mean (±SD) sleepiness score on the Epworth Sleepiness Scale was 8.9 ± 4.3 with 37.6% of respondents scoring higher than 10, reflecting excessive daytime sleepiness. Most of the study participants (73.9%) had a strategy to manage sleep preceding an ultramarathon, with 54.7% trying to increase their opportunities for sleep. Only 21% of participants reported that they had a strategy to manage sleep during ultramarathons, with micronaps being the most common strategy specified. Sub-analyses from 221 responses indicated that sleep duration during an ultramarathon was correlated with finish time for races lasting 36–60 h (r = 0.48; p < 0.01) or > 60 h (r = 0.44; p < 0.001). We conclude that sleep duration among ultramarathon runners was comparable to the general population and other athletic populations, yet they reported a lower prevalence of sleep disorders. Daytime sleepiness was among the lowest rates encountered in athletic populations, which may be related to the high percentage of nappers in our population. Sleep extension, by increasing sleep time at night and daytime napping, was the main sleep strategy to prepare for ultramarathons.

## Introduction

Sleep quality and sleep wake cycle characteristics are underappreciated factors that can affect sport performance [1–3]. Like other body functions, physiological and psychomotor functions involved in exercise have a circadian rhythmicity, leading to a diurnal variation in sport performance (for a review, see [2]). Sleep and sport/exercise have a strong relationship, mutually influencing each other, positively or negatively. Sleep deprivation is associated with higher rating of perceived effort (RPE) values, potentially leading to reduced performance, particularly in endurance event (e.g.[4,5]). It has also been suggested that sleep deprivation leads to a higher rate of injury [6], reduces muscle glycogen stores [7] and alters recovery after muscle damage induced by exercise [8]. Acute sleep deprivation and chronic sleep restriction also induce alterations in glucose metabolism, with increases in insulin resistance and decreased insulin sensitivity [9]. Both also impact different aspects of cognitive performance [10] such as increased reaction time and lapses during attentional tasks [11,12] and psychomotor functions [13]. Sleep deprivation and disruption of circadian rhythms also lead to elevated levels of inflammatory markers such as interleukins (IL-6, IL 10) and tumor necrosis factor (TNF-α), and alter immune status [14,15]. The elevation of inflammatory markers, especially IL-6, has been associated with increased pain ratings in response to sleep restriction [16]. In contrast, good sleep, as well as sleep extension strategies, can enhance performance by preventing the decrease in cognitive performance and reducing the RPE during exercise [11,17].

An ultramarathon is a category of long distance running longer that the traditional marathon of 42.195 km, with races being of specific distances ranging from 50 km to 100 miles (161 km, e.g. Western States Endurance Run, Ultra-trail^®^ du Mont Blanc^®^ (UTMB^®^)) or even longer (e.g., Tor des Geants^®^: 335 km), specific durations (24 h to 6 days) or in stages over multiple days (e.g. Marathon des Sables). Ultramarathons, particularly those run on trails, have become more and more popular [18]. Associated with this has been a growing interest in research related to ultramarathon running [19]. Until now however, research has mostly focused on factors that impact performance (like pain, gastro-intestinal distress or physiological determinants) (e.g. [18]), health consequences of the elevated training load of ultramarathon runners [20], the medical issues and management of common injuries and illnesses encountered in ultramarathons [21,22] and more specific issues such as exercise-associated muscle cramping [23], exercise-associated hyponatremia [24] and fatigue/biomarkers changes (e.g. [25,26]) during ultramarathons.

Ultramarathon participation requires high training loads and long duration training sessions, leading to high recovery needs, particularly relative to sleep. Consistent sleep of 7–9 h per night is recommended for healthy adults [27], but some authors suggest that athletes should sleep between 9 and 10 h per night to allow sufficient recovery [28]. Moreover, some ultramarathons are long enough that runners are required to maintain their effort for durations longer than their usual wakefulness period, with nocturnal activity and brief, or sometimes no, opportunities for sleep. Presumably, poor sleep habits and/or inadequate (or lack of) appropriate sleep strategies before a race can potentially exacerbate fatigue, and increase risk of injury, hallucinations and failure to finish.

To the best of our knowledge, only one study, conducted by Poussel et al. [29] on 303 finishers of the UTMB^®^, has focused on how ultramarathon runners manage sleep before and sleepiness during an ultramarathon. The study used a questionnaire that was completed after the 2013 UTMB^®^, a race in which the winning time is about 21 hours and runners are allowed up to 46 hours to complete, so it involves one or two nights of sleep deprivation. Before the race, 88% of runners reported that they adopted specific sleep management strategies such as naps, increased sleep time during the previous nights, or training in sleep deprivation. Most finishers (72%) reported that they did not sleep at all during the race, and not surprisingly, these runners finished faster than those who slept. Race time was positively correlated with drowsiness. Interestingly, runners who increased sleep time before the race as a pre-race strategy also completed the race faster. This observation is in line with studies showing the effects of prophylactic naps on vigilance (e.g [30]).

Thus, the first purpose of the present study was to further describe the habitual sleep characteristics and strategies of ultramarathon runners relative to their intensity of training. We included several components of their habitual lifestyle that might influence sleep behaviors such as time of training, use of stimulants and napping, as well as the presence of sleep disorders. The second purpose was to examine strategies used by runners to manage sleep before and during ultramarathons. This descriptive analysis may be of practical importance for ultramarathon runners and coaches relative to preparation and performance and can also serve as baseline information for future intervention studies on sleep and performance.

## Methods

### Procedure

Ultramarathon runners were invited to complete a questionnaire through electronic mailings, postings on various ultramarathon-related web sites and forums, and advertisements in magazines related to ultramarathon running in France, Italy and the US. Conditions for participating were a minimum age of 18 yrs and having completed an ultramarathon at some time in the past. All participants completed a secure anonymous web-based questionnaire (Google Survey) that included demographic questions, in addition to questions related to sleep, medical history and training history. The questionnaire was offered in French, Italian and English. All procedures were approved by the University of Calgary Conjoint Health Research Ethics Board.

The questionnaire inquired about independent variables, corresponding to subject characteristics such as age, weight, height, history of training and competition in ultramarathons. It also examined various behaviors that might influence sleep (time of day when training was performed), sleep habits (sleep duration during weekdays, weekends and holidays), use of naps, and history of sleep disorders (difficulty falling or remaining asleep, use of sleep medication and medical assistance with sleep problems). The questionnaire also included the Epworth Sleepiness Scale (ESS) [31,32]. The ESS is a self-administered questionnaire used to investigate excessive daytime sleepiness that has a high level of internal consistency as measured by Cronbach's alpha (0.88). Scores on the ESS can range from 0 to 24, and a score above 10 is regarded as an indicator of excessive sleepiness. Additionally, ESS scores of 0–5 indicate low normal daytime sleepiness, 6–10 indicate high normal daytime sleepiness, 11–12 indicate mild excessive daytime sleepiness, 13–15 indicate moderate excessive daytime sleepiness, and 16–24 indicate severe excessive daytime sleepiness.

Participants were also asked if and how they have been attentive to their sleep in the days and nights preceding an ultramarathon and if and how they manage sleep during ultramarathons. When they indicated they had a strategy, they were invited to describe the type of strategy used. In case of a mismatch in the provided answers (e.g. if the runner answered “yes” to the question “did you sleep during the race?” then wrote “I did not sleep”), the written response was considered to be correct. For those with a sleep strategy during an “overnight ultramarathon” and a “longer than 2 night ultramarathon”, we requested the participant’s sleep duration (in minutes) and the race finish time (in hours). If respondents provided information for several race experiences, we considered each of them. If an answer did not contain both sleep duration and race finish time, the response was not considered for analysis. We also considered the number of sleep episodes, the location of the sleep episodes (aid station, building, etc. vs outside, i.e. on the trail) and the time of day for the sleep episodes.

### Statistical analysis

Descriptive statistics are presented. The data are reported as mean and SD since they passed normality testing, performed using the Skewness-Kurtosis test. Missing data are noted where pertinent. Normality allowed us to use Generalized Estimating Equations (GEE) to compare sleep durations during weekdays, weekends and holidays [generalized linear model and generalized estimating equations (GENLIN) procedure], due to the correlated nature of observations from the same participant. Other associations between variables were assessed using a Chi-square test, with phi (ϕ) value to report the effect size. Correlations between sleep duration during a race and race finish time were determined using Pearson correlation analyses. Statistical analysis was performed with SPSS (24.0) and statistical significance was set at p < 0.05.

## Results

### Population

Out of the 636 participants, 393 responded in French, 118 in Italian and 125 in English. They consisted of 541 (85.1%) men and 95 (14.9%) women. Mean (± SD) height and weight were 177.8 ± 8.5 cm and 72.3 ± 8.4 kg for men and 165.2 ± 24.6 cm and 57.2 ± 8.1 kg for women, respectively. Other subject characteristics are shown in Table 1.

**Table 1:** Selected characteristics of the subjects.

	n	%
**Age (yrs)**		
18–29	66	10.4
30–39	198	31.1
40–49	244	38.4
50–59	104	16.4
> 60	24	3.8
		
**Number of ultra-marathons per year**		
< 1	72	11.3
1	180	28.3
2	184	28.9
3	112	17.6
4	48	7.5
≥ 5	40	6.3
		
**Hours of training per week during normal training periods**		
< 3	23	3.6
3–6	212	33.3
6–9	239	37.6
9–12	118	18.6
12–15	29	4.6
> 15	15	2.4
		
**Hours of training per week during intense training periods**		
<3	4	0.6
3–6	40	6.3
6–9	149	23.4
9–12	196	30.8
12–15	153	24.1
> 15	94	14.8

### Sleep characteristics

#### Sleep duration

**Fig 1:**
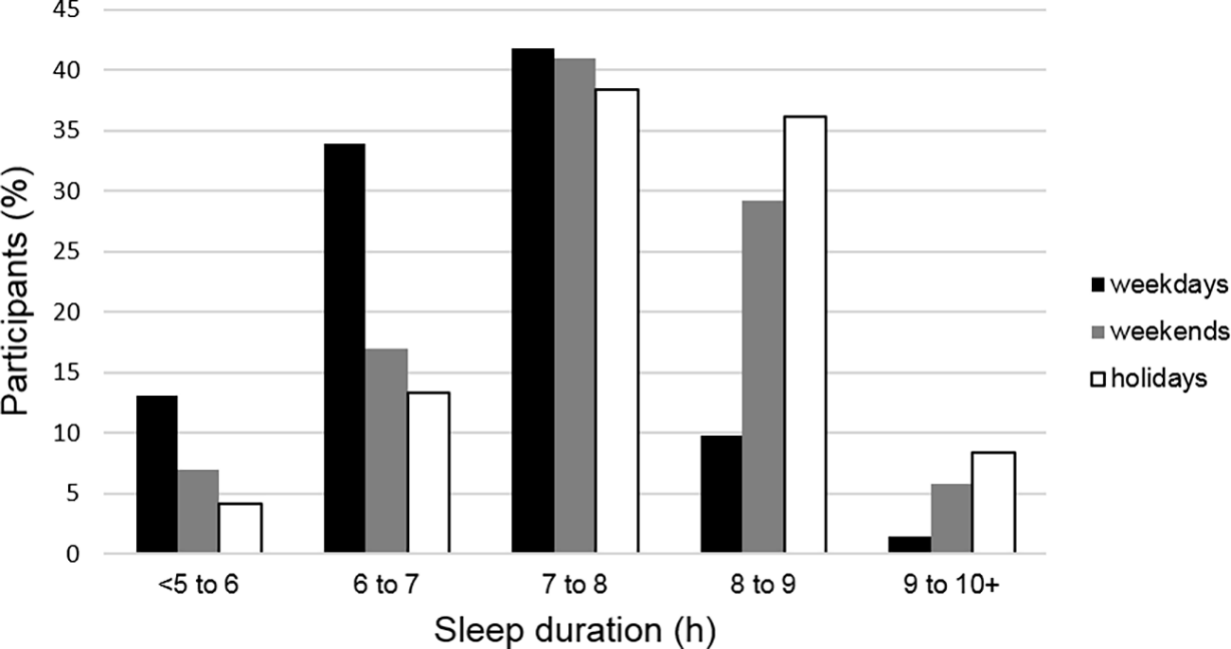
Habitual sleep patterns for weekdays, weekends and holidays.

GEE results indicate that there was a significant effect (χ2(2) = 516.683, p < 0.001) of condition (weekdays, weekends, holidays) on sleep duration. Multiple comparisons indicate that these three periods were all significantly different from each other (p < 0.001). During weekdays, sleep durations of 6–7 h and 7–8 h per night were reported most frequently. During weekends and holidays, sleep durations of 7–8 h and 8–9 h per night were reported the most (Fig 1).

#### Napping

**Fig 2:**
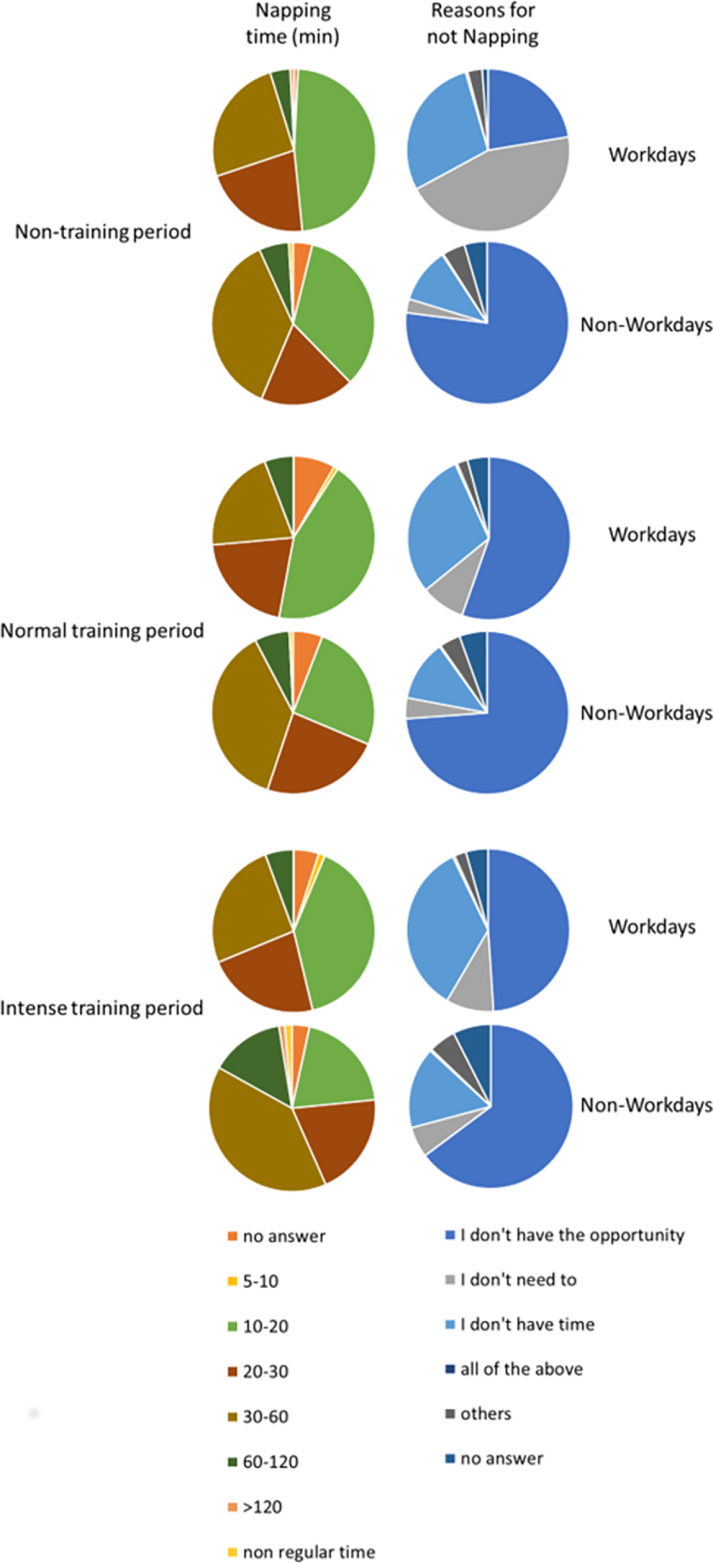
Distribution of napping durations and reasons for not napping during the different training periods and during workdays and non-workdays.

Key results related to nap habits are displayed in Fig 2. As expected, the percentage of nappers was lower during working days. Indeed, on working days only 19.8%, 19.0% and 25.2% napped during non-training, normal training and intense training periods, respectively. During non-working days, the percentage of nappers was 37.1%, 42.9% and 55.8% for the same periods. During non-working days, participants mostly indicated they napped for 10–20 min (33.9%) and 30–60 min (36.9%) during non-training periods, and most of them indicated they took naps of 30–60 min (37.4%) during normal training periods. During intense training periods, 39.7% reported napping for 30 to 60 min, 20% between 10 and 20 min and 20% between 20 and 30 min.

The main reasons for not napping in all conditions (working vs non-working and training vs non-training periods) was either a lack of opportunity (up to 77.0%), or a lack of time (up to 45.0%). One exception was for non-training periods on work days, in which the largest percentage of participants indicated they did not need to nap (44.7%).

#### Sleep disturbances

There were 24.5% (n = 156) of the participants reporting trouble falling asleep or waking during the night. Of those reporting such sleep disturbances, 22.4% (n = 35) had sought medical assistance about their sleep problems and 14.1% (n = 22) used medicine or a medical device to sleep. Medications included zolpidem, benzodiazepines, tricyclic antidepressants, zopiclone, homeotherapy and phytotherapy, hypnotics, antiemetics and melatonin.

Chi square tests did not reveal (p > 0.05) a link between presence of sleep problems and age category, or time of day when training was performed. However, there was a significant link between sex and sleep disorders (φ = 0.14; p < 0.005), with more women (38.9%) reporting sleep problems than men (22.0%).

**Fig 3:**
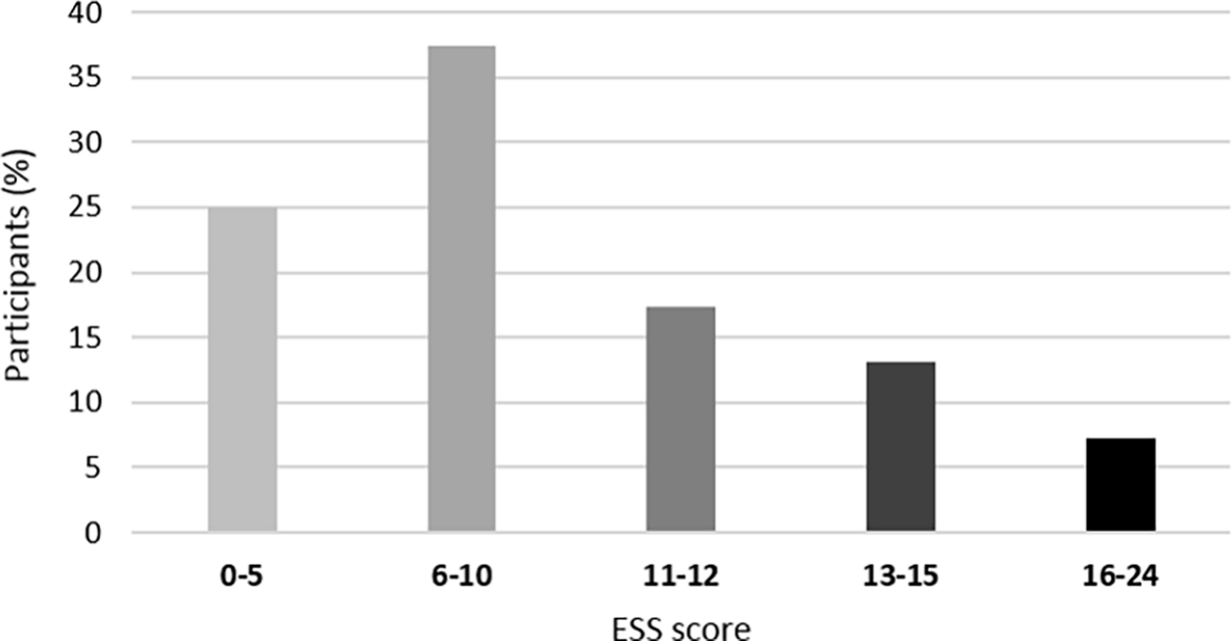
Percentage of participants with each ESS score range.

Mean (± SD) ESS score was 8.9 ± 4.3. Over a third (37.6%) of participants had an ESS score higher than 10, reflecting excessive daytime sleepiness, and 7.2% had a score between 16 and 24, reflecting a severe excessive daytime sleepiness (Fig 3).

#### Behaviors that may affect sleep-wake cycle

The time of day when training was performed (allowing multiple answers) was reported as being “at the beginning of the evening (6 PM—8 PM)” by 41.2%, “in the morning (7:00 AM -12:00 PM)” by 38.8%, and between 12 PM and 2 PM by 25.6% of participants. In the context of sleep, it is interesting to note that 95 runners (14.9%) reported they train “in the evening (after 8:00 PM)”, and 135 (21.2%) reported training ‘‘before 7:00 AM”.

Participants were also asked about the use of substances susceptible to affecting sleep (Table 2). First, 96.1% were non-smokers, yet some respondents indicated they smoked more than 10 cigarettes/day. Concerning consumption of stimulants, 53% reported consuming 1–3 cups (25 cl) of coffee per day, 20% reported consuming 4–6 cups per day and 23.3% indicated they did not consume coffee. One to 3 cups a day of tea was consumed by 36.6% of respondents, whereas 57% did not consumed tea. Only 17.1% of participants reported drinking soda containing caffeine. Finally, 64.7% of participants indicated they did not drink alcohol, and 28.4% reported drinking 1–3 glasses of alcoholic beverages per day.

**Table 2:** Use of substances affecting sleep.

	Cigarettes (number/day)		Tea (cups/day)		Coffee (cups/day)		Caffeinated soda (cans or bottles/day)		Alcohol (glasses/day)	
	n	%	n	%	n	%	n	%	n	%
**0**	611	96.1	362	56.9	148	23.3	526	82.8	411	64.6
**< 1**	0	0.0	10	1.6	6	0.9	14	2.2	37	5.8
**1 to 3**	9	1.4	233	36.6	337	53.0	89	14.0	181	28.5
**4 to 6**	9	1.4	25	3.9	127	20.0	5	0.8	7	1.1
**7 to 10**	3	0.5	5	0.8	12	1.9	1	0.2	0	0.0
**> 10**	4	0.6	1	0.2	6	0.9	0	0.0	0	0.0

#### Sleep strategies before ultramarathons

Most of the study participants (73.9%) reported that they are attentive to their sleep in the days and nights preceding an ultramarathon. Strategies to enhance sleep are shown in Table 3 with sleep extension being the most common strategy.

**Table 3:** Sleep strategies used during the days and nights preceding an ultramarathon among subjects indicating they have pre-race sleep strategies. Most respondents selected multiple responses.

	n	%
Sleep accumulation: earlier bed time and/or later rise time in the morning (even if no need to sleep)	257	54.7
Good sleep habits: regular sleep time; keeping habitual sleep time; focus on natural need to sleep	119	25.3
Increased napping time	94	20.0
Sleep accumulation at night and increased diurnal napping time	68	14.5
Other: relaxation/avoid stress; isolation; food changes; matching sleep habits with race start time; decreased training load; “rest”	39	8.3
Adapt work schedule/take days off	17	3.6
Avoid stimulants	14	3.0
Sleep medication	12	2.6
Avoid screens at night	7	1.5

#### Sleep strategies during ultramarathons

For ultramarathon races lasting through one night, 75 out of 456 runners (16.4%) reported sleeping during the event. There were 120 out of 216 runners (55.6%) who reported sleeping during a race that lasted through 2 nights, and 88 of 93 (94.6%) reported sleeping when competing in races comprising more than 2 nights. Only 21% of participants reported that they have a strategy to manage sleep during ultramarathons. The frequency of use of various strategies is shown in Table 4, with micronaps being the most common strategy specified.

**Table 4:** Sleep strategies used during ultramarathons among subjects indicating they had slept during an ultramarathon.

	n	%
Micronap < 5 min	6	4.5
Nap 5–10 min	4	3.0
Nap 10–20 min	9	6.7
Nap 20–30 min	10	7.5
Nap 30–60 min	2	1.5
Sleep episode > 1 h	18	13.4
Nap (unspecified duration)	19	14.2
Micronap + sleep episode > 1 h	3	2.2
Sleep when exhausted	12	9.0
Resist pressure to sleep (stimulant use)	17	12.7
Delay to next aid station	5	3.7
Relaxation	4	3.0
Not specified	25	18.7

Micronap was defined as a nap of 5 min or less; nap was defined as a sleep episode of 5 to 60 min. Micronap + sleep episode was defined as the use of short naps at some points in the race and at least one sleep episode > 1 h.

**Fig 4:**
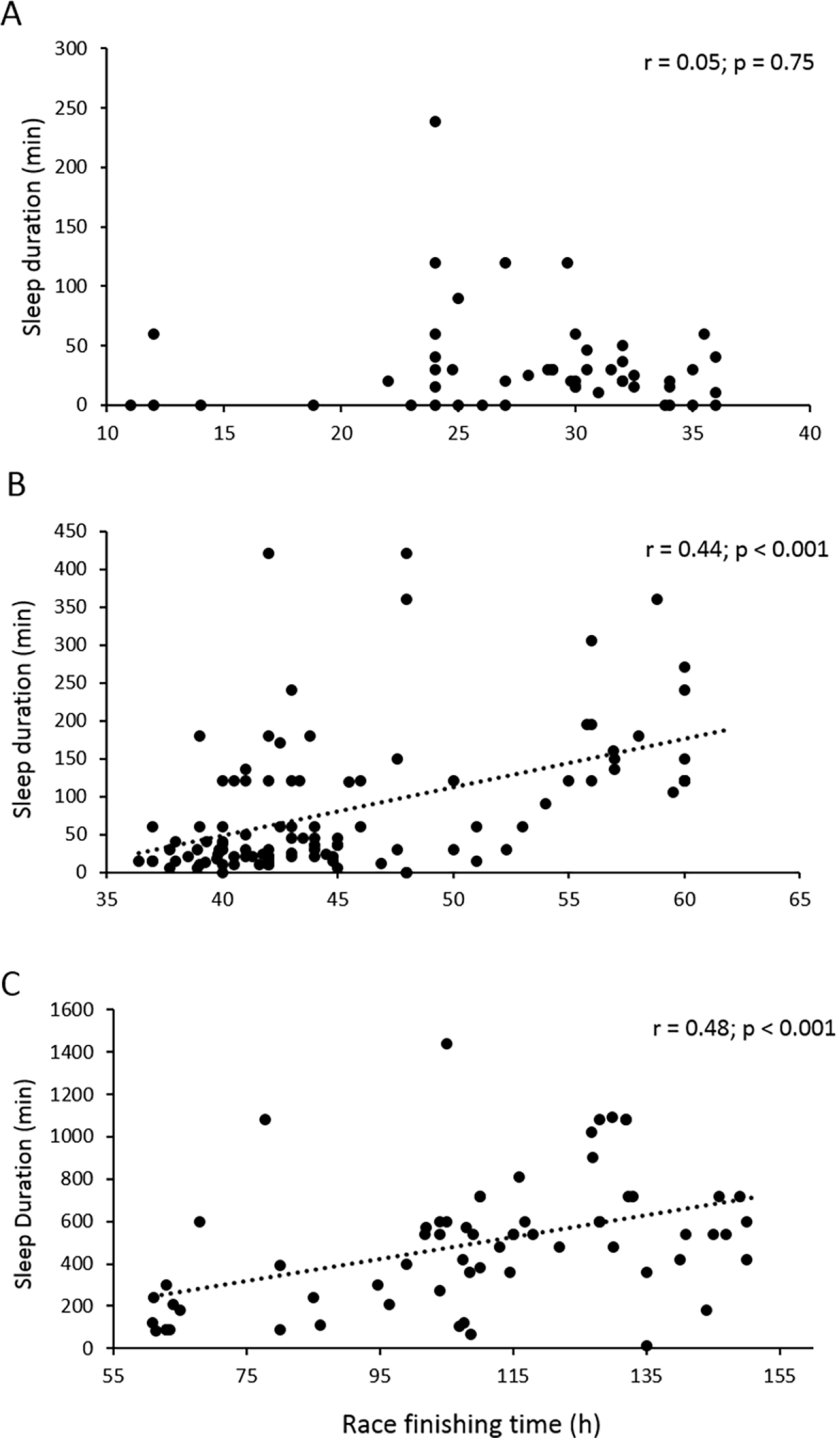
Correlations between sleep duration and race time for races shorter than 36 h (panel A), races lasting between 36 and 60 h (panel B) and races longer than 60 h (panel C). No correlation was found between sleep duration and finish time for races ≤ 36 h (r = -0.05; p = 0.75), yet sleep duration and finish time were correlated for races lasting 36–60 h (r = 0.48; p < 0.01) or > 60 h (r = 0.44; p < 0.001).

We also analyzed sleep duration and number of sleep episodes as a function of race finish time in the 221 responses provided by the runners. Considering races with finish times ≤ 36h (mean ± SD finish time = 27.9± 6.2 h), 36–60 h (45.4 ± 6.8 h) and > 60 h (109.0 ± 26.3 h), sleep durations were 0.55 ± 0.70 h, 1.36 ± 1.51 h and 8.24 ± 5.15 h, respectively. Relationships between sleep time and race finish time are shown in Fig 4. The mean (± SD) number of sleep episodes was 1 ± 1, 2 ± 3, 6 ± 3 for races with finish times < 36 h, 36–60 h and > 60 h, respectively. Only a small subset of participants (n = 24) indicated the time of day when naps or periods of sleep took place. Among those, the mean (± SD) time of day was 11:34 ± 5:36 PM. About 75% of the participants slept between 11:00 PM and 7:00 AM and 25% slept between 7:00 AM and 11:00 PM.

## Discussion

The aims of the present study were to (i) characterize sleep habits and behaviors related to sleep among ultramarathon runners, and (ii) describe sleep management by runners before and during ultramarathons. Key findings were that this population of ultramarathon runners had sleep durations comparable to the general population, with longer sleep durations during weekends and holidays than weekdays, and a lower prevalence of sleep disorders than the general population. We also noticed a high percentage of nappers in our population, especially in non-working periods. Finally, in preparation for ultramarathon events, runners considered sleep extension as the main sleep strategy to prepare for ultramarathons.

### Demographic data

These results were obtained from 636 participants from different countries, with 14.9% being women. This is a lower percentage of women compared with previous large surveys. For instance, 32% of respondents were women in the Ultrarunners Longitudinal TRAcking (ULTRA) study [20,33], and international ultramarathon race statistic from 2017 indicate that 20.1% of finishers were women [34]. Yet it is a higher percentage than at the UTMB^®^ (< 10%). Our population consisted largely of runners who were 30–39 yrs or 40–49 yrs of age which was in accordance with the mean age of participants in previous ultramarathon studies of around 40 yrs [18,20,23,33,35], and international ultramarathon race statistics shown that 31% and 33.3% are 30–40 and 40–50 yrs of age, respectively [34]. Thus, our study population appears to have been an appropriate representation of ultramarathon runners with regards to sex and age.

### Sleep characteristics

Most ultramarathon runners in the present study reported that they sleep 6 to 8 h per night during weekdays and 7 to 9 h per night during weekends and holidays, which fits with recent data on healthy adults in which sleep was reported to average 6.85 hours on weekdays and 7.62 hours on non-workdays according to a nationwide survey in the US in 2013 [36]. This also corresponds to the values found in an European population, i.e. 7–9 h of sleep per night [37,38]. Leeder et al. [39] reported an actual mean (±SD) sleep time of 6.9 ± 0.7 h in athletes vs. 7.2 ± 0.4 h for a non-sporting control group, whereas athletes spent 8.6 ± 0.9 h in bed, vs. 8.1 ± 0.3 h for the non-sporting control group. Lastella et al. [40] reported a total sleep time ranging from 6.1 to 7.1 h in individual sports (cycling, mountain bike, race walking, triathlon and swimming) [41] and a total mean (±SD) sleep time of 7.4 ± 0.6 h in endurance cyclists. Unfortunately, these studies did not specify if the data were obtained for weekdays, weekends or both. As in the general population, ultramarathon runners tend to increase sleep duration during weekends rather than reducing sleep in order to have more training time.

An interesting result of the present study is that despite taking naps during the day, only ~25% of the respondents reported trouble falling asleep or waking during the night, which appears less than rates (31% to 46%) reported in the general population of Western European countries [42,43]. A recent national survey conducted by the American National Sleep Foundation [36] indicated that exercisers are significantly less likely to have sleep disorders than non-exercisers. Since longer durations of exercise have been linked with greater benefits from exercise [44], it is reasonable to postulate that the type of training performed by ultramarathon runners would be effective at improving sleep quality [45,46]. It must also be noted that, due to large differences in the characterization of sleep disorders or problems, comparison across studies is difficult. For example, Ohayon et al. [47] examined the incidence of insomnia without symptoms, which was higher (48%) than in the present study and the “dissatisfaction with sleep quantity or quality”, which was close or even lower than in the present study (from 6.8 to 29.9%). This makes the comparison with our assessment of sleep problems difficult. The present finding of sleep disorders being more common in women than men is consistent with previous studies investigating the epidemiology of sleep disorders [42,47,48]. However, the percentage of women with sleep disorders appeared higher in our study (38.9%) compared with the studies cited above, which is difficult to explain.

The percentage of ultramarathon runners with sleep problems who had sought medical advice (22.4%) and taken medicine to sleep (14.1%) was lower than what has been observed in previous surveys in the Western population [42] indicating that 53% of individuals with sleep problems have spoken to a physician and 50% have received a drug prescription. Zolpidem and benzodiazepines were the most cited sleep medications used by the present sample of ultramarathon runners, which is in accordance with previous data in athletes (see [49–51] for reviews).

### Napping

The present survey revealed that less than half of the ultramarathon runners take naps, but as anticipated, work was an important mediator of napping habits. This high prevalence may be related to the higher daytime sleepiness in our sample than in the general population, probably due to high level of fatigue associated with training load. While 19–25% reported napping on work days, depending on their level of training, 37–56% reported napping on non-work days. Consistent with our findings, a previous report of exercisers (23–60 years of age) observed less napping during workdays (30–34% of respondents) than non-work days (40–45% of respondents) [36]. Yet the percentage of nappers in our study appears to be higher than what was reported in another study (15%) among participants of individual sports involving heavy training loads such as cycling, mountain biking, race walking, swimming and triathlon [41].

### Daytime sleepiness

The ultramarathon runners examined herewith had a mean ESS score of 8.9, which was higher than scores observed in general Western populations, i.e. 7.9 in Germany [52], 6.9 in Norway [53], 6.1 to 8.3 in the US [54]. Moreover, 37.6% of our sample had an ESS score higher than the classical cut off score of 10, reflecting excessive daytime sleepiness. This percentage was also higher than normative values found in the general population, ranging from 10.8 to 30.4% [53,55–57]. High sleepiness scores (> 6) in athletes is not a surprising observation since previous studies in several athletic populations recorded mean ESS scores similar to the present study (ranging from 8.2 to 8.5) in hockey, cricket, soccer players, cyclists and triathletes [58]. On the contrary, ESS scores were found to be 12.5 and 9.6 in collegiate tennis players [59] and basketball players [28], respectively.

Athletes who are balancing high training loads and long duration training sessions with other life demands, coupled with a need for more sleep from the high levels of training, may receive inadequate sleep, leading to higher sleepiness during the day [60], despite the high rate of napping observed in the present study. Daytime sleepiness is a multifactorial phenomenon, induced by behavioral practices (late night initiation of sleep, light exposure at night, early waking) or internal disorders such as circadian rhythm desynchronization [61]. Given that regular exercisers usually display robust circadian rhythms [62], it can be hypothesized that daytime sleepiness in this group is largely related to inadequate sleep duration. Another possibility is that some of these athletes are overtrained, since it is known that overtraining can increase daytime sleepiness score [63].

### Behaviors that may affect the sleep-wake cycle

A relatively high proportion (> 35%) of the study participants reported that they train in the early morning (before 7:00 AM) or in the evening (after 8:00PM). We can suspect that the early or late training is generally used to limit disruption of professional, social and personal (family) demands, and it likely adversely affects sleep duration. Furthermore, exercise late in the day can affect sleep quality. Exercise between 8 to 3 h before habitual bedtime has been thought to be beneficial for sleep, since it could advance the phase of melatonin and promote sleep onset [64]. Moreover, exercise in the evening can facilitate sleep through increased peripheral skin blood flow following the initial hyperthermia from exercise, which decreases core temperature and in turn favors sleep [65,66]. Yet, exercising less than 3 h before bed time may disrupt sleep onset and phase delay the melatonin excretion [67]. However, several studies have not found disrupted sleep from training at night or late evening [68–70]. In addition, a recent meta-analysis did not reach a consensus concerning “good timing” of exercise for sleep [44].

High doses of caffeine (600 mg, i.e. 5–6 cup) [71–73], nicotine consumption [74] and alcohol consumption [75] are known to have an acute disruptive effect on sleep. For instance, caffeine and nicotine increase sleep latency, decrease sleep efficiency and sleep duration, induce more frequent awakening, and change sleep architecture (decrease slow wave sleep, increase REM sleep latency). It is not surprising that avoiding stimulants was reported as a strategy to optimize the duration and quality of sleep before races.

### Strategies used to manage sleep before and during ultramarathons

#### Sleep strategies before an ultramarathon

Seventy-four percent of the runners reported being attentive to sleep preceding an ultramarathon, a value slightly lower than in the only study that has examined this question [29], where 88% of the runners reported adopting a sleep strategy before the race. In the present study, sleep extension was achieved by 55% of runners through increasing nighttime sleep duration and by 20% through the use of daytime napping. Poussel et al. [29] also have reported the increase of daytime napping as a strategy to prepare an ultramarathon, but the rate of runners using this strategy was higher (41%) than in our study. Increased time of napping close to a competition is a way to complement night sleep and has been previously described by Sargent et al. [76] in elite athletes. Of note is the fact that 14.5% of the present subjects reported they increased both time in bed and diurnal napping time before the race.

In a study of collegiate basketball players [28], it was suggested that sleep extension could improve physical performance, though the study was limited by lack of a control group. Moreover, it has been shown that sleep extension can protect against the effects of sleep deprivation, mainly at the cognitive level [11,77]. For instance, we showed that 6 nights of sleep extension prevented both reaction time degradation during a psychomotor vigilance test and the number of microsleep episodes during sleep deprivation [11]. In the same protocol, we also showed that sleep extension before a night of total sleep deprivation improved time to exhaustion during a lower-limb sustained isometric contraction [17]. The beneficial effect on performance was likely due to the reduced RPE after sleep deprivation when preceded by sleep banking (increasing sleep opportunity at night) since cortical voluntary activation (assessed by transcranial magnetic stimulation) was not different between the sleep extension and the control conditions [4]. Although this needs to be confirmed in further studies, it can be speculated that the longer the exercise, the greater the benefits that can be derived from sleep banking because the role of perceptual responses is probably more important in prolonged exercise [78]. Thus, sleep accumulation before an ultramarathon seems to be a good strategy to minimize the cognitive and perceptual effects of sleep deprivation and exercise.

In addition to sleep banking, another highly cited sleep strategy (~25%) among the present study participants was to utilize good sleep habits (regularity of sleep/wake times or paying attention to natural need of sleep). Regularity of sleep timing is associated with greater subjective and objective sleep quality [79,80] and lower daytime sleepiness [81]. As previously demonstrated in elite athletes, strategies such as establishing a regular bedtime, avoiding the consumption of alcohol and caffeine and increasing napping can optimize the duration and quality of sleep in athletes [40,41].

#### Sleep strategies during an ultramarathon

As expected, the percentage of ultramarathon runners sleeping during a race was found to depend on the duration of the race, with less than 20% sleeping for a race lasting through one night and almost all runners sleeping for multiple days races. In the study of UTMB^®^ finishers with a mean (± SD) race finish time of 39.5 ± 5.1 h, Poussel et al. [29] reported that 28% of the runners did not sleep, and non-sleepers were significantly faster than those who slept. From the present 221 runner subsample, we found a mean (± SD) cumulated sleep duration of 0.55 ± 0.70 h, 1.36 ± 1.51 h and 8.24 ± 5.15 h for races < 36 h, between 36 and 60 h, and longer than 60 h, respectively. Such an amount of sleep, even if it could decrease the risk of accident due to sleep deprivation, is enough to have an important effect on finish time, especially among top runners.

The majority of runners who indicated “having a strategy of sleep management” indicated they take one or several naps. When nap duration was specified, most runners reported that naps ranged from 10 to 20 min (6.7%) and 20 to 30 min (7.5%). Naps between 10 and 15 min (5%) and between 15 and 30 min (11%) were also the most cited by Poussel et al. [29]. In the present study, sleep was taken mainly in aid stations. Some participants also indicated they would wait until the aid station to sleep, even if sleepiness was elevated. The perspective of reaching an aid station could be a motivation for participants to continue the race and postpone their sleep. A few respondents (n = 24) also provided the time when sleep occurred. While a small sample, it is interesting to note that about 75% of the runners reported they slept at night, which is the normal time during the 24-hour day for rest in humans, since sleep pressure is higher and the timing of the circadian system promotes sleepiness [82].

### Study limitations

The present study has some limitations. One limitation relates to recall bias, linking to inaccuracy or absence of answers regarding sleep duration and finish time of races. Another issue is that it is appropriate to emphasize that our study population was a convenience sample that may not be fully representative of the population of ultramarathon runners. Moreover, results on sleep duration relative to race time was derived from a subsample of only 221 responses with some individuals providing multiple responses. We also acknowledge that the percentage of women participants in our study was slightly lower than in the general population of ultramarathon runners. Finally, we acknowledge a potential limitation with our assessment of sleep disorders, which was done through simply asking if they had trouble falling asleep or waking during the night, if they had visited a physician for a sleep problem, and if they had taken medication to sleep. While these questions may only provide a limited insight into a sleep disorder, they focus on the most common complains about sleep disorders (i.e. difficulty falling asleep and waking up at night) and the management strategy. Those performing future studies should consider a more elaborate assessment that includes questions beyond insomnia (e.g. related to other common disorders such as sleep apnea and restless legs syndrome and inclusion of a scale like the insomnia severity scale.

## Conclusions

In conclusion, the ultramarathon runners in this study had sleep durations comparable to the general population and other athletic populations, yet they reported a lower prevalence of sleep disorders. Daytime sleepiness, while higher than normative values found in the general population, was among the lower rates encountered in athletic populations, which may be related to the high percentage of nappers in our population. Before ultramarathon races, 55% of the ultramarathon runners considered sleep extension as the main sleep strategy to prepare for ultramarathons, largely accomplished through increasing sleep time at night. Finally, sleeping during races was correlated with race duration for races longer than 36 h. Whether short naps or longer sleep episodes (e.g. one sleep cycle) is the most effective strategy in terms of performance in extremely long ultramarathons remains to be assessed. We suggest that future studies use actigraphy to provide more precise measurement of sleep before and during races. We also encourage the scientific community to examine the effects of sleep extension on ultramarathon performance.

## Supporting information

S1 File Questions and answer provided by all participants. (XLSX)

S2 File Questionnaire provided to the participant in English, French and Italian Languages. (DOCX)
