# Supplementary material for: Sleep habits and strategies of ultramarathon runners
Source: PLoS One. 2018 May 9;13(5):e0194705. doi: 10.1371/journal.pone.0194705 (PMC5942705; doi:10.1371/journal.pone.0194705)
Supplement: S2 File — (DOCX) [file pone.0194705.s002.docx]

**Sleep habits and strategies of ultramarathon runners**

**Sleep habits and strategies of ultramarathon runners-Questionnaire**

**English version**

**Gender**

- Male
- Female

**Height**

**Weight**

**What is your occupation?**

**Where do you live?**

**Age**

- 18-29
- 30-39
- 40-49
- 50-59
- 60

**When do you usually work?**

- During the day (between 7 am and 8 pm)
- Shift work
- At night
- Unemployed

**When do you usually train?**

- Before 7 am
- In the morning (7 am -12 pm),
- In the afternoon (2 pm - 6 pm)
- At the beginning of the evening (6 pm - 8 pm)
- In the evening (after 8PM)
- It changes every day

**During normal training, how many hours a week do you train?**

- <3 hours
- 3 to 6 hours
- 6 to 9 hours
- 9 to 12 hours
- 12 to 15 hours
- More than 15 hours

**During periods of intense training, how many hours a week do you train?**

- <3 hours
- 3 to 6 hours
- 6 to 9 hours
- 9 to 12 hours
- 12 to 15 hours
- More than 15 hours

**How many ultramarathons races do you participate in every year?**

**Have you ever visited a physician for a sleep problem?**

- Yes
- No

**Do you have trouble falling asleep or do you wake during the night?**

- Yes
- No

**Do you usually take medicine to sleep?**

- Yes
- No

**If so, which drugs do you take?**

**Which methods do you use to fall asleep?**

**How many cigarettes do you smoke per day?**

**How many cups of coffee do you drink per day?**

**How many cups of tea do you drink per day?**

**How many glasses of Coke (or Pepsi) do you drink per day?**

**How many glasses of alcohol do you drink per day?**

**How many hours do you sleep per night during the week when you work?**

- less than 5
- Between 5 and 6
- Between 6 and 7
- Between 8 and 9
- Between 7 and 8
- Between 9 and 10
- more than 10

**How many hours do you sleep per night during the week-end when you work during the week?**

- less than 5
- Between 5 and 6
- Between 6 and 7
- Between 8 and 9
- Between 7 and 8
- Between 9 and 10
- more than 10

**How many hours do you sleep per night when you are on vacation?**

- less than 5
- Between 5 and 6
- Between 6 and 7
- Between 8 and 9
- Between 7 and 8
- Between 9 and 10
- more than 10

**Do you take a nap when you do not train and you work during the day?**

- Yes
- No

**If not, why not?**

- I don't have the opportunity
- I don't need to
- I don't have time
- all of the above
- other:

**If so, what is its average duration?**

**Do you take a nap when you do not train and you do not work?**

- Yes
- No

**If not, why not?**

- I don't have the opportunity
- I don't need to
- I don't have time
- all of the above
- other:

**If so, what is its average duration?**

**Do you take a nap when you train normally and you work during the day?**

- Yes
- No

**If not, why not?**

- I don't have the opportunity
- I don't need to
- I don't have time
- all of the above
- other:

**If so, what is its average duration?**

**Do you take a nap when you train normally and you don't twork?**

- Yes
- No

**If not, why not?**

- I don't have the opportunity
- I don't need to
- I don't have time
- all of the above
- Autre :

**If so, what is its average duration?**

**Do you take a nap when you train a lot and you work during the day?**

- Yes
- No

**If not, why not?**

- I don't have the opportunity
- I don't need to
- I don't have time
- all of the above
- others:

**If so, what is its average duration?**

**Do you take a nap when you train a lot and you don't work?**

- Yes
- No

**If not, why not?**

- I don't have the opportunity
- I don't need to
- I don't have time
- all of the above
- others

**If so, what is its average duration?**

**Have you performed an overnight ultra-trail?**

- Yes
- No

**If so, did you sleep?**

- Yes
- No

**If so, how and when?**

**Have you performed an ultra-trail comprising 2 nights of racing?**

- Yes
- No

**If so, did you sleep?**

- Yes
- No

**If so, how and when?**

**Have you performed an ultra-trail comprising more than 2 nights of racing?**

- Yes
- No

**If so, did you sleep?**

- Yes
- No

**If so, how and when?**

**Do you have a particular strategy of sleep management during ultras?**

- Yes
- No

**If so, what is this strategy?**

**Do you pay particularly attention to your sleep in the days and nights preceding an ultra?**

- Yes
- No

**If so, what do you do?**

**Epworth sleepiness scale**

How likely are you to doze off or fall asleep in the following situations, in contrast to feeling just tired? This refers to your usual way of life in recent times. Even if you haven’t done some of these things recently try to work out how they would have affected you.

Use the following scale to choose the most appropriate number for each situation:

0 = would never doze

1 = slight chance of dozing

2 = moderate chance of dozing

3 = high chance of dozing

It is important that you answer each question as best you can.

Situation #1: Sitting and Reading

Would never doze

Slight chance of dozing

Moderate chance of dozing

High chance of dozing

Situation #2: Watching TV

Would never doze

Slight chance of dozing

Moderate chance of dozing

High chance of dozing

Situation #3: Sitting, inactive in a public place (e.g. a theater or a meeting)

Would never doze

Slight chance of dozing

Moderate chance of dozing

High chance of dozing

Situation #4: As a passenger in a car for an hour without a break

Would never doze

Slight chance of dozing

Moderate chance of dozing

High chance of dozing

Situation #5: Lying down to rest in the afternoon when circumstances permit

Would never doze

Slight chance of dozing

Moderate chance of dozing

High chance of dozing

Situation #6: Sitting and talking to someone

Would never doze

Slight chance of dozing

Moderate chance of dozing

High chance of dozing

Situation #7: Sitting quietly after lunch without alcohol

Would never doze

Slight chance of dozing

Moderate chance of dozing

High chance of dozing

Situation #8: In a car, while stopped for a few minutes in traffic

Would never doze

Slight chance of dozing

Moderate chance of dozing

High chance of dozing

**Sleep habits and strategies of ultramarathon runners**

**Sleep habits and strategies of ultramarathon runners-Questionnaire**

**French version**

**Quelle est votre sexe ?**

- homme
- femme

**Quelle est votre taille ?**

**Quel est votre poids ?**

**Quelle est votre profession ?**

**Où habitez-vous?**

**Quel est votre âge ?**

- 18-29
- 30-39
- 40-49
- 50-59
- 60

**Quels sont vos horaires de travail habituels ?**

- Le jour (entre 7H et 20H)
- Posté
- De nuit
- Sans emploi

**Quels sont vos horaires d'entraînement habituels ?**

- Avant 7H
- Le matin (7H-12H)
- In the afternoon (2 pm - 6 pm)
- En début de soirée (18H-20H)
- Le soir (après 20H)
- Cela varie chaque jour

**En période d’entrainement normal, combien d’heures d’entraînement faites-vous par semaine ?**

- <3 heures
- 3 à 6 heures
- 6 à 9 heures
- 9 à 12 heures
- 12 à 15 heures
- Plus de 15 heures

**En période d’entrainement intense, combien d’heures d’entraînement faites-vous par semaine ?**

- 3 heures
- 3 à 6 heures
- 6 à 9 heures
- 9 à 12 heures
- 12 à 15 heures
- Plus de 15 heures

**Combien d'ultra-trails (> 80 km) faites-vous habituellement par an ?**

**Avez-vous déjà consulté un médecin pour un problème de sommeil ?**

- Oui
- Non

**Avez-vous des problèmes particuliers d’endormissement ou de réveils nocturnes ?**

- Oui
- Non

**Prenez-vous habituellement des médicaments pour dormir ?**

- Oui
- Non

**Si oui, de quel(s) médicament(s) s’agit-il ?**

**Quelles méthodes utilisez-vous pour dormir ?**

**Combien fumez-vous de cigarettes par jour en moyenne ?**

**Combien de tasses de café buvez-vous par jour en moyenne ?**

**Combien de tasses de thé buvez-vous par jour en moyenne ?**

**Combien de verres de Coca-cola (ou Pepsi-cola) buvez-vous par jour en moyenne ?**

**Combien de verres d'alcool buvez-vous par jour en moyenne ?**

**Combien d’heures en moyenne dormez-vous en semaine lorsque vous travaillez ?**

- Moins de 5
- entre 5 et 6
- Entre 6 et 7
- Entre 8 et 9
- Entre 7 et 8
- Entre 9 et 10
- Plus de 10

**Combien d’heures en moyenne dormez-vous le week-end lorsque vous travaillez pendant la semaine ?**

- Moins de 5
- Entre 5 et 6
- Entre 6 et 7
- Entre 8 et 9
- Entre 7 et 8
- Entre 9 et 10
- Plus de 10

**Combien d’heures en moyenne dormez-vous lorsque vous êtes en vacances ?**

- Moins de 5
- Entre 5 et 6
- Entre 6 et 7
- Entre 8 et 9
- Entre 7 et 8
- Entre 9 et 10
- Moins de 10

**Faites-vous la sieste, lorsque vous ne vous entraînez pas et que vous travaillez ?**

- Oui
- Non

**Si non, pourquoi**

- Je n'ai pas la possibilité matérielle de la faire
- Je n'en ressens pas le besoin
- Je n'ai pas le temps
- Chacune des raisons ci-dessus
- Autres

**Si oui, quelle est sa durée moyenne ?**

**Faites-vous la sieste, lorsque vous ne vous entraînez pas et que vous ne travaillez pas ?**

- Oui
- Non

**Si non, pourquoi ?**

- Je n'ai pas la possibilité matérielle de la faire
- Je n'en ressens pas le besoin
- Je n'ai pas le temps
- Chacune des raisons ci-dessus
- Autres

**Si oui, quelle est sa durée moyenne ?**

**Faites-vous la sieste, lorsque vous vous entraînez normalement et que vous travaillez ?**

- Oui
- Non

**Si non, pourquoi ?**

- Je n'ai pas la possibilité matérielle de la faire
- Je n'en ressens pas le besoin
- Je n'ai pas le temps
- Chacune des raisons ci-dessus
- Autres

**Si oui, quelle est sa durée moyenne ?**

**Faites-vous la sieste, lorsque vous vous entraînez normalement et que vous ne travaillez pas ?**

- Oui
- Non

**Si non, pourquoi ?**

- Je n'ai pas la possibilité matérielle de la faire
- Je n'en ressens pas le besoin
- Je n'ai pas le temps
- Chacune des raisons ci-dessus
- Autres

**Si oui, quelle est sa durée moyenne ?**

**Faites-vous la sieste, lorsque vous vous entraînez intensément et que vous travaillez ?**

- Oui
- Non

**Si non, pourquoi ?**

- Je n'ai pas la possibilité matérielle de la faire
- Je n'en ressens pas le besoin
- Je n'ai pas le temps
- Chacune des raisons ci-dessus
- Autres

**Si oui, quelle est sa durée moyenne ?**

**Faites-vous la sieste, lorsque vous vous entraînez intensément et que vous ne travaillez pas ?**

- Oui
- Non

**Si non, pourquoi ?**

- Je n'ai pas la possibilité matérielle de la faire
- Je n'en ressens pas le besoin
- Je n'ai pas le temps
- Chacune des raisons ci-dessus
- Autres

**Si oui, quelle est sa durée moyenne ?**

**Avez-vous déjà réalisé un ultra-trail comprenant une nuit en course ?**

- Oui
- Non

**Si oui, avez-vous dormi ?**

- Oui
- Non

**Si oui, comment et quand ?**

**Avez-vous déjà réalisé un ultra-trail comprenant 2 nuits en course ?**

- Oui
- Non

**Si oui, avez-vous dormi ?**

- Oui
- Non

**Si oui, comment et quand ?**

**Avez-vous déjà réalisé un ultra-trail comprenant plus de 2 nuits en course ?**

- Oui
- Non

**Si oui, avez-vous dormi ?**

- Oui
- Non

**Si oui, comment et quand ?**

**Avez-vous une stratégie particulière de gestion du sommeil pendant un ultra ?**

- Oui
- Non

**Si oui, quelle est-elle ?**

**Accordez-vous un soin particulier au sommeil les jours et les nuits précédents un ultra-trail ?**

- Oui
- Non

**Si oui, que mettez-vous en place ?**

**Echelle de Somnolence d’Epworth**

Vous arrive-t-il de somnoler ou de vous endormir (dans la journée) dans les situations suivantes : Même si vous ne vous êtes pas trouvé récemment dans l'une de ces situations, essayez d'imaginer comment vous réagiriez et quelles seraient vos chances d'assoupissement.

Situation n°1 : Assis(e) en train de lire

Aucune chance de somnoler

Faible chance de s’endormir

Chance modérée de s’endormir

Forte chance de s’endormir

Situation n°2 : En train de regarder la télévision

Aucune chance de somnoler

Faible chance de s’endormir

Chance modérée de s’endormir

Forte chance de s’endormir

Situation n°3 : Assis(e), inactif(e) dans un lieu public (théâtre, cinéma, réunion…)

Aucune chance de somnoler

Faible chance de s’endormir

Chance modérée de s’endormir

Forte chance de s’endormir

Situation n°4 : Comme passager(e) d’une voiture (ou transport en commun) roulant sans arrêt pendant une heure

Aucune chance de somnoler

Faible chance de s’endormir

Chance modérée de s’endormir

Forte chance de s’endormir

Situation n°5 : Allongé(e) l’après-midi pour vous reposer, lorsque les circonstances le permettent

Aucune chance de somnoler

Faible chance de s’endormir

Chance modérée de s’endormir

Forte chance de s’endormir

Situation n°6 : Etant assis(e) en train de parler avec quelqu’un

Aucune chance de somnoler

Faible chance de s’endormir

Chance modérée de s’endormir

Forte chance de s’endormir

Situation n°7 : Assis(e) au calme après un repas sans alcool

Aucune chance de somnoler

Faible chance de s’endormir

Chance modérée de s’endormir

Forte chance de s’endormir

Situation n°8 : Dans une voiture immobilisée depuis quelques minutes

Aucune chance de somnoler

Faible chance de s’endormir

Chance modérée de s’endormir

Forte chance de s’endormir

**Sleep habits and strategies of ultramarathon runners**

**Sleep habits and strategies of ultramarathon runners-Questionnaire**

**Italian version**

**Qual’è il tuo sesso?**

- Uomo
- Donna

**Quanto sei alto/a ?**

**Quanto pesi ?**

**Qual’è la tua professione ?**

**Dove abiti ?**

**Quanti hai ?**

- 18-29
- 30-39
- 40-49
- 50-59
- 60

**Qual’è il tuo orario di lavoro abituale ?**

- Diurno (tra le 7:00 e le 20:00)
- A turni
- Di notte
- Disoccupato

**Quali sono i tuoi orari abituali di allenamento ?**

- Prima delle 7:00
- Mattino (tra le 7:00 e le 12:00)
- Tra le 12:00 e le 18:00
- Prima serata (tra le 18:00 et le 20:00)
- La sera (dopo 20:00)
- Varia di giorno in giorno

**Normalmente qual’è la durata complessiva dei tuoi allenamenti (su base settimanale)**

- Meno di 3 ore
- da 3 a 6 ore
- da 6 a 9 ore
- da 9 a 12 ore
- da 12 a15 ore
- Più di 15 ore

**Durante i periodi di allenamento intenso qual’è la durata complessiva di allenamento?(su base settimanale)**

- Meno di 3 ore
- da 3 a 6 ore
- da 6 a 9 ore
- da 9 a 12 ore
- da 12 a15 ore
- Più di 15 ore

**Abitualmente, a quante competizioni di ultra-trail (> 80 km) partecipi ogni anno ??**

**Hai già consultato un medico per problemi di sonno ??**

- Si
- No

**Hai difficoltà ad addormentarti, hai problemi di risveglio notturno ?**

- Si
- No

**Prendi medicinali per dormire ?**

- Si
- No

**Se sì, di quali medicinali si tratta ?**

**Quali metodi utilizzi per addormentarti ?**

**Mediamente quante sigarette fumi al giorno ?**

**Mediamente quante tazze di caffè bevi al giorno ?**

**Mediamente quante tazze di tè bevi al giorno ?**

**Mediamente quanti bicchieri di Coca-cola (o Pepsi-cola) bevi al giorno ?**

**Mediamente quanti bicchieri di bevande alcoliche bevi al giorno ?**

**Mediamente quante ore dormi durante una normale settimana lavorativa ?**

- Meno di 5 ore
- Tra le 5 e le 6 ore
- Tra le 6 e le 7 ore
- Tra le 7 e le 8 ore
- Tra le 8 e le 9 ore
- Tra le 9 e le 10 ore
- Più di 10 ore

**Mediamente quante ore dormi durante il week-end di una normale settimana lavorativa ?**

- Meno di 5 ore
- Tra le 5 e le 6 ore
- Tra le 6 e le 7 ore
- Tra le 7 e le 8 ore
- Tra le 8 e le 9 ore
- Tra le 9 e le 10 ore
- Più di 10 ore

**Mediamente quante ore dormi quando sei in vacanza ?**

- Meno di 5 ore
- Tra le 5 e le 6 ore
- Tra le 6 e le 7 ore
- Tra le 7 e le 8 ore
- Tra le 8 e le 9 ore
- Tra le 9 e le 10 ore
- Più di 10 ore

**Fai una siesta quando lavori e non ti alleni ?**

- Si
- No

**Se non la fai, perchè ?**

- Non posso farla (assenza di condizioni che me lo permettano)
- Non ne sento il bisogno
- Non ho tempo
- tutti i precedenti
- altro:

**Se la fai, qual’è la durata media della siesta ?**

**Fai la siesta se non lavori e non ti alleni?**

- Si
- No

**Se non la fai, perchè ?**

- Non posso farla (assenza di condizioni che me lo permettano)
- Non ne sento il bisogno
- Non ho tempo
- tutti i precedenti
- altro:

**Se la fai, qual’è la durata media della siesta ?**

**Fai la siesta se lavori e ti alleni normalmente ?**

- Si
- No

**Se non la fai, perchè ?**

- Non posso farla (assenza di condizioni che me lo permettano)
- Non ne sento il bisogno
- Non ho tempo
- tutti i precedenti
- altro:

**Se la fai, qual’è la durata media della siesta ?**

**Fai la siesta se non lavori e ti alleni normalmente ?**

- Si
- No

**Se non la fai, perchè ?**

- Non posso farla (assenza di condizioni che me lo permettano)
- Non ne sento il bisogno
- Non ho tempo
- tutti i precedenti
- altro:

**Se la fai, qual’è la durata media della siesta ?**

**Fai la siesta se lavori e ti alleni intensamente ?**

- Si
- No

**Se non la fai, perchè ?**

- Non posso farla (assenza di condizioni che me lo permettano)
- Non ne sento il bisogno
- Non ho tempo
- tutti i precedenti
- altro:

**Se la fai, qual’è la durata media della siesta ?**

**Fai la siesta se non lavori e ti alleni intensamente ?**

- Si
- No

**Se non la fai, perchè ?**

- Non posso farla (assenza di condizioni che me lo permettano)
- Non ne sento il bisogno
- Non ho tempo
- tutti i precedenti
- altro:

**Se la fai, qual’è la durata media della siesta ?**

**Hai già terminato un ultra-trail che comprende una notte di gara ?**

- Si
- No

**Se si, hai dormito ?**

- Si
- No

**Se si, come e quando ?**

**Hai già concluso un Ultra-trail che include 2 notti di gara ?**

- Si
- No

**Se si, hai dormito ?**

- Si
- No

**Se si, come e quando ?**

**Hai già concluso un Ultra-trail che include più di 2 notti di gara ?**

- Si
- No

**Se si, hai dormito ?**

- Si
- No

**Se si, come e quando ?**

**Avez-vous une stratégie particulière de gestion du sommeil pendant un ultr** **Hai una strategia particolare per la gestione del sonno durante un Ultra ?**

- Si
- No

**Se si, qual’è la strategia ?**

**Attribuisci un’importanza particolare al sonno nei giorni che precedono e seguono un ultra-trail ?**

- Si
- No

**Se si, segui qualche precauzione particolare ?**

**Epworth Sleepiness Scale**

Che probabilità ha di appisolarsi o di addormentarsi nelle seguenti situazioni, indipendentemente dalla sensazione di stanchezza? La domanda si riferisce alle usuali abitudini di vita nell'ultimo periodo. Qualora non si sia trovato di recente in alcune delle situazioni elencate sotto, provi ad immaginare come si sentirebbe

Situazione n°1 : seduto/a, durante la lettura nessuna probabilità di addormentarmi

minima probabilità di addormentarmi

moderata probabilità di addormentarmi

forte probabilità di addormentarmi

Situazione n°2 : seduto/a, guardando la televisione

nessuna probabilità di addormentarmi

minima probabilità di addormentarmi

moderata probabilità di addormentarmi

forte probabilità di addormentarmi

Situazione n°3 : seduto/a, inattivo/a in un luogo pubblico(teatro, cinema, riunione)

nessuna probabilità di addormentarmi

minima probabilità di addormentarmi

moderata probabilità di addormentarmi

forte probabilità di addormentarmi

Situazione n°4 : viaggiando per più di un’ora, come passeggero/a in auto o su un mezzo pubblico

nessuna probabilità di addormentarmi

minima probabilità di addormentarmi

moderata probabilità di addormentarmi

forte probabilità di addormentarmi

Situazione n°5 : disteso/a per il riposo, durante il pomeriggio e con condizioni che lo permettono

nessuna probabilità di addormentarmi

minima probabilità di addormentarmi

moderata probabilità di addormentarmi

forte probabilità di addormentarmi

Situazione n°6 : seduto/a, in treno, conversando con qualcuno

nessuna probabilità di addormentarmi

minima probabilità di addormentarmi

moderata probabilità di addormentarmi

forte probabilità di addormentarmi

Situazione n°7 : seduto/a, dopo un pasto senza aver bevuto alcolici

nessuna probabilità di addormentarmi

minima probabilità di addormentarmi

moderata probabilità di addormentarmi

forte probabilità di addormentarmi

Situazione n°8 : in auto, durante una sosta di durata superiore a qualche minuto

nessuna probabilità di addormentarmi

minima probabilità di addormentarmi

moderata probabilità di addormentarmi

forte probabilità di addormentarmi
